# Genomic Position Mapping Discrepancies of Commercial SNP Chips

**DOI:** 10.1371/journal.pone.0031025

**Published:** 2012-02-17

**Authors:** João Fadista, Christian Bendixen

**Affiliations:** 1 Department of Clinical Sciences Malmö, CRC, Lund University, Malmö, Sweden; 2 Department of Molecular Biology and Genetics, Faculty of Science and Technology, Aarhus University, Tjele, Denmark; Emory University School Of Medicine, United States of America

## Abstract

The field of genetics has come to rely heavily on commercial genotyping arrays and accompanying annotations for insights into genotype-phenotype associations. However, in order to avoid errors and false leads, it is imperative that the annotation of SNP chromosomal positions is accurate and unambiguous. We report on genomic positional discrepancies of various SNP chips for human, cattle and mouse species, and discuss their causes and consequences.

## Introduction

Array based genotyping provide a powerful tool to interrogate genetic variation. It enables a broad variety of applications such as genome-wide association studies, evaluation of genetic merit in breeding applications, linkage disequilibrium studies, comparative genetic studies, as well as for characterizing biodiversity [1]. High density genotyping arrays are produced for a number of species having a reference genome available, and the SNPs on the arrays are mapped to genomic positions. For convenience and standardization the mapping data is distributed along with the arrays to the end user. Apart from crude positional information, the mapping position provides the basis for annotation of potential effects of the SNP alleles upon RNA splicing, regulatory elements or amino acid exchanges. Furthermore, the relative order of the SNPs may in some cases effect data analysis and generation of haplotypes, imputation as well as linkage studies. We here report that minor SNP positional discrepancies exist in various Affymetrix™ and Illumina™ genotyping arrays made for human, mouse and cow species, and discuss the possible functional consequences.

## Results

To detect genomic position discrepancies of SNPs in genotyping arrays, we used BLAST [2] with highly sensitive parameter settings, and restricted the analysis to examine only SNPs that mapped with a unique perfect match to the species genome in question (Methods).

In order to explain possible differences in mapping we chose to compare first our mappings of the BovineSNP50 v1 beadchip (http://www.illumina.com/products/bovine_snp50_whole-genome_genotyping_kits.ilmn) with the ones made by Illumina™. From the 54,001 SNPs present on the chip, we found that only 41,496 (77%) had a unique and perfect match in both our and Illumina™ mappings (Data S1). Illumina™ reports the unique mapping of 52,255 SNPs while we mapped 10% less (46,760) using the same genome assembly (Bt4.0). The difference can be explained by the fact that Illumina™ did not use the unassembled chromosome (ChrUn, which consists of almost 10% of genomic sequence of the assembly) as part of their mapping process. Using 41,496 uniquely mapped SNPs by both and omitting mapping differences if they were on ChrUn, we detected 99 SNP genomic position discrepancies, of which 16 (17%) are in different genes. These 99 differences lead to a change in genomic order of 7,209 SNPs, with 99% being less or equal than 2 indexes away (Data S1).

Next, we examined the discrepancies between our mappings and the ones made by a study that previously reported mappings differences relative to the Illumina™ BovineSNP50 [3]. As before, we used SNPs found to be uniquely mapped in both studies. From 41,536 SNPs, we detected 764 differences (Data S1). By manually checking a fraction of those alignments we noted that, albeit their mapping procedure is correct, their post-processing script leads to calling SNPs 1 bp upstream or downstream of the actual SNP genomic position (Data S1). It should be noted that while we used BLAST [2] in the present study the previous report used MEGABLAST [4]. Using a shorter word size (9 versus 28), our search is likely to result in a better alignment sensitivity (a mapping file with the updated SNP positions is supplied as Data S1).

Recently, a new version of the BovineSNP50 beadchip (v2) came into the market which includes 54,609 SNPs in comparison to 54,001 SNPs from the previous version. Using the same procedure we mapped the SNP postions for this beadchip using only the SNPs that have a unique perfect hit in the genome assembly (UMD3.1 in this case). From 48,284 SNPs, we detected 449 SNP genomic position discrepancies, of which 248 (55%) were in different genes. These 449 discrepancies lead to a change in the genomic order of 13,133 SNPs, with 90% being less or equal than 2 index positions away (updated mapping file provided as Data S2).

Having identified discrepancies in the mapping of Illumina's™ BovineSNP50 beadchips, we decided to study a number of other high-density genotyping arrays. First, we analyzed the BovineHD beadchip (http://www.illumina.com/products/bovinehd_whole-genome_genotyping_kits.ilmn), and found that only 14 SNPs (2 in different chromosomes) retrieved mapping to different genomic positions, of which 2 (14%) were observed in different genes. These 14 discrepancies lead to a change in genomic order of 182 SNPs, with 98% being less or equal than 1 index away (Data S3). By manually checking these 14 mapping discrepancies, we found three reasons for the Illumina™ mismapping: (1) presence of an extra SNP on the SNP flanking sequence, (2) or having less flanking sequence aligned, or (3) mapped to 1 bp apart near the actual SNP position.

Next, we analyzed the Affymetrix™ mouse diversity genotyping array (http://media.affymetrix.com/support/technical/datasheets/mouse_diversity_array_datasheet.pdf). We detected that 620 SNPs (5 in different chromosomes) retrieved different genomic mapping positions, of which 66 (11%) are in different genes. These 620 differences lead to a change in genomic order of 271,325 SNPs, with 98% being less or equal than 2 indexes away (Data S4). By manually checking a fraction of these discrepancies, we found that those Affymetrix™ mismappings are either due to their hit not being perfect, or having one or more extra SNPs on the SNP flanking sequence.

Our next step was to detect SNP position discrepancies in two of the most widely used genotyping arrays in human studies: Illumina's™ Human1M-Duo DNA Analysis beadchip (http://www.illumina.com/products/human1m_duo_dna_analysis_beadchip_kits.ilmn) and the Affymetrix's™ Genome-Wide Human SNP Array 6.0 (http://media.affymetrix.com/support/technical/datasheets/genomewide_snp6_datasheet.pdf). Concerning the Affymetrix™ human genotyping array, we detected 25 differences (5 in different chromosomes), of which 10 (40%) were in different genes. These 25 discrepancies lead to 61,916 SNPs being in a different genomic order, with 89% being less or equal than 2 indexes away (Data S5). By checking manually a subset of those alignment discrepancies, we found that the Affymetrix™ mismappings were due to their hit not being perfect, having one or more extra SNPs on the SNP flanking sequence, or having less flanking sequence aligned. With the Illumina's™ human beadchip, 271 SNPs have different genomic positions (with 22 on different chromosomes), of which 59 (22%) are on different genes. The 271 discrepancies lead to 131,378 SNPs being in a different genomic order, although 98% are less or equal than two indexes away (Data S6). By checking manually a subset of those alignment discrepancies, we found that the Illumina™ mismappings were due to their hit not being perfect, having one or more extra SNPs on the SNP flanking sequence, or having less flanking sequence aligned.

**Figure 1:**
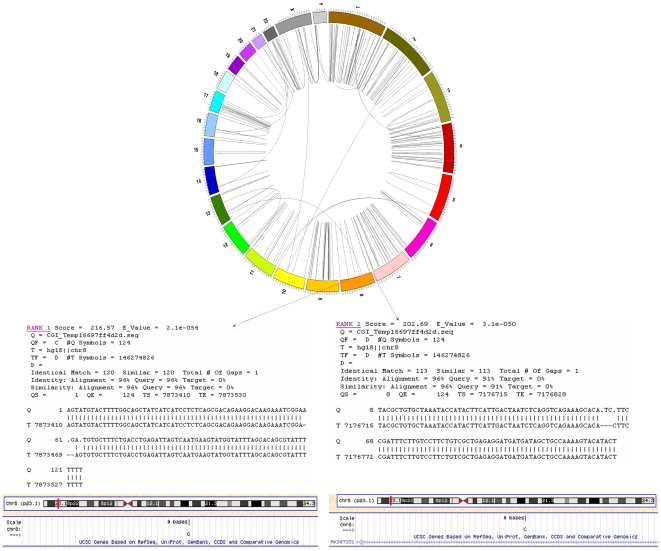
Example of a wrongly mapped SNP found significant in a GWAS (Macgregor et al. 2010). The red SNP shown in the circular human karyotype diagram instead of being mapped to position 7,873,470 in chr 8 (Rank1), it is wrongly mapped by Illumina™ to position 7,176,768 in chr8 (Rank2), with less 7 bp of the SNP flanking sequence aligned.

Furthermore, we were interested in a more detailed understanding of the possible functional impact of relying on incorrectly mapped positions. Therefore, we searched through a selection of papers published in 2010 for SNPs associated with various human phenotypes and found that two of the SNPs with positional discrepancies on the Illumina's™ Human1M-Duo DNA Analysis beadchip were reported to be significantly correlated with human traits in recent genome wide association studies [5]–[7]. The first, rs2523608, has a positional discrepancy of only 1 bp but since it is in an intronic region of a gene it might be enough to trigger different an erroneous prediction functional consequences. It is shown to be significant in two papers from 2010 related to HIV [5]–[6]. The second, rs9692809, has a positional discrepancy of 697 kb and does overlap a hypothetical gene in the Illumina™ mapping but not in ours. It is shown to be significantly associated with vertical optic cup-to-disc ratio [7]. Figure 1 shows the alignment of this SNP by both Illumina™ and our own mapping data.

## Discussion

In summary, despite most SNPs map reliably and consistently and most changes in position are relatively localized, the SNP discrepancies found in this report clearly suggest that more sensitive parameters of the aligners (whether they are BLAST, MegaBLAST or other) should be used in order to achieve an accurate chromosomal alignment instead of retrieving a partial best alignment with extra SNPs, indels or less SNP flanking sequence aligned (Table 1).

**Table 1:** Summary of the chromosomal SNP position discrepancies for each SNP genotyping platform tested.

Platform	Total SNPs	diff position	diff chr	diff Index (only in same Chr)	SNPs in diff genes
Affymetrix Mouse Diversity Array	576,284	620	5	271,325 (96%≤2 index away)	66 (11%)
Affymetrix Human SNP Array 6.0	934,968	25	5	61,916 (89%≤2 index away)	10 (40%)
Illumina Human1M-Duo BeadChip	1,163,218	271	22	131,378 (99%≤2 index away)	59 (22%)
Illumina BovineHD Beadchip	775,003	14	2	182 (98%≤1 index away)	2 (14%)
Illumina BovineSNP50 v1 Beadchip	52,255	99	0	7,209 (99%≤2 index away)	16 (17%)
Illumina BovineSNP50 v2 Beadchip	54,060	449	3	13,133 (90%≤2 index away)	248 (55%)

Since wrongly mapped SNPs can change in which genic and regulatory regions they overlap, it can trigger erroneous variant effect conclusions. Large SNP positional discrepancies can also affect studies of genotype imputation and linkage disequilibrium, leading to false coverage and power of genome-wide association analysis and erroneous evaluation of the choice of SNP platform to use [8]. Our study here do not intend to cast doubt on the main conclusions of any paper, but rather intend to ensure that future studies use the correct chromosomal SNP positions in order to minimize erroneous conclusions.

We would recommend the providers of commercial SNP chips to always provide (for each chip) a technical report on how they exactly did the mapping. Specifically, refer to which mapping algorithm and its parameters used, genome assembly version, and the location of SNP flanking sequences in their websites. It was our experience that trying to retrieve this information revealed to be a cumbersome task, with little or no information provided regarding the SNP mapping procedure.

In Supplementary data we provide our mappings for the genotyping platforms tested here, and we hope that investigators using different genotyping platforms are encouraged to map them using an accurate and sensitive procedure (Methods). The SNPs that map to multiple regions can also be easily retrieved from public databases such as dbSNP, UCSC or Ensembl. These SNPs most probably map to paralogous regions of the genome with high sequence identity [9]–[10].

## Methods

### Gathering the data

All SNP discrepancies reported here are relatively to the genome build to which the chip was initially mapped to. The fasta files for the genome assemblies of each species queried were retrieved from ftp://hgdownload.cse.ucsc.edu/goldenPath/mm9/bigZips/chromFa.tar.gz (mouse assembly mm9), ftp://hgdownload.cse.ucsc.edu/goldenPath/hg18/bigZips/chromFa.zip (human assembly hg18), ftp://ftp.cbcb.umd.edu/pub/data/assembly/Bos_taurus/Bos_taurus_UMD_3.1/bos_taurus.fa.gz (cow assembly UMD3.1) and ftp://hgdownload.cse.ucsc.edu/goldenPath/bosTau4/bigZips/bosTau4.fa.gz (cow assembly BosTau4.0).

The cow genome has currently two genome versions available, one (Btau4.0) from the public consortium that sequenced the bovine genome [11], and other (UMD3.1) from University of Maryland Steven Salzbergs's group [12]. Despite claims that UMD3.1 is better than Btau4.0 [12], we decided to use both assemblies because some of the chips tested here were mapped by Illumina to Btau4.0 (BovineSNP50 v1 beadchip) and others to UMD3.1 (BovineSNP50 v2 and BovineHD beadchips).

The genomic coordinates of each SNP and the fasta files for the oligomer sequences flanking the SNPs in each chip were taken from different sources.For the Illumina™ arrays, these data were fetched from ftp://ftp.illumina.com/Whole%20Genome%20Genotyping%20Files/. This site is only accessible through password that can be provided by Illumina™ customer services. Inside this folder there are subfolders containing the four type of arrays tested here (subfolders BOVINEHD_Product_Files, BOVINESNP50VERSION1_product_files, BovineSNP50VERSION2_product_files and Human1M-Duo_v3_product_files). The files used were BovineHD_B.csv, BovineHD_777962_Name_Chr_Coord.csv, BovineSNP50_B.csv, BovineSNP50_Final_SNPs_54001.csv, BovineSNP50v2_AlleleReport_revB, BovineSNP50v2_FinalSNPList_54609_09Apr10.csv, Human1M-Duov3_B_csv, Human1M-Duov3_FinalMarkerList_1199187.txt.

For the Affymetrix™ arrays, the data was fetched from http://www.affymetrix.com/browse/products.jsp?productId=131533&navMode=34000&navAction=jump&aId=productsNav#1_3 and from http://www.affymetrix.com/estore/browse/products.jspjsessionid=07BF945B0A18133EA55E7EE9D965B154?productId=prod100002&categoryId=cat30008#1_3. The files used were GenomeWideSNP_6.bed, GenomeWideSNP_6_flanking_sequences_fasta and MOUSEDIVm520650.na31.annot.csv. Contrary to the human array, the Affymetrix™ website for the mouse array did not contain the flanking sequences for the respective SNPs. After contacting Affymetrix™ support, they told us that The Mouse Diversity Genotyping array was not designed by Affymetrix, but by The Jackson Laboratory and the University of North Carolina. As such, they suggested us to take a look at the website of the Jackson Laboratory (http://cgd.jax.org/tools/diversityarray.shtml) to see whether we could find the flanking sequences files. Unfortunately we were not able to get it and therefore we retrieved this information from dbSNP mouse build 129 (http://www.ncbi.nlm.nih.gov/projects/SNP/), where the SNP flanking information was stored. Consequently, for this SNP chip we did not get all the data from the primary source but from a secondary source which might add to the reasons for this array having the biggest number of diverging SNP positions.

“The gene annotations were retrieved from UCSC genome browser (http://genome.ucsc.edu/). For human and mouse, the gene track ‘UCSC Genes’ table ‘knownGene’ was used, while for cow the gene track ‘Ensembl Genes’ table ‘ensGene’ was used for assembly Btau 4.0 and the file ftp://ftp.cbcb.umd.edu/pub/data/assembly/Bos_taurus/Bos_taurus_UMD_3.1/annotation/UMD3.1.gff.gz was used for assembly UMD3.1.”The NHGRI catalog of GWAS studies (http://www.genome.gov/gwastudies/) was used to select papers published in 2010 from which significant SNPs were detected to have mapping discrepancies.

### Alignment

The alignment process was done with a hardware accelerated version of BLAST [2] called TeraBlast™ [13]. This algorithm aligns the oligomer sequences flanking the SNPs of each genotyping chip at higher speed than if it was performed with the standard version of BLAST. TeraBlast™ was run with the following parameters:

[WORD SIZE] 9
[QUERY INCREMENT] 3
[EXTENSION THRESHOLD] 20
[EXPECTATION] 0.00000001
[QUERY FORMAT] FASTA/PEARSON
[TARGET FRAMES] D
[Comment] Following line selects query both complement and direct
[QUERY SEARCH] B
[THRESHOLD] 20
[MAX SCORES] 3
[MAX ALIGNMENTS] 3
[NUCLEIC MATCH] 1
[NUCLEIC MISMATCH] -3
[Comment] VALUES FOR GAPPED ALIGNMENT: BANDED (NEW) T (FULL SW ALIGN) OR F
[GAPPED ALIGNMENT] T
[OPEN PENALTY] -2
[EXTEND PENALTY] -1

It should be noted that the performance speed depends on the WORD SIZE and QUERY INCREMENT, with lower word size and lower query increment increasing the sensitivity of the alignment.

### Post-processing

The aligned output files were formatted with Unix commands. After, the genomic coordinates of each perfectly unique mapped SNP were compared with the original genomic coordinates annotated by Affymetrix™ and Illumina™. This was done with custom R scripts. Both Unix and R commands are provided in Data S7.

## Supporting Information

Data S1 **BovineSNP50 v1 beadchip uniquely mapped SNPs (all and discordant).**
(RAR)

Data S2 **BovineSNP50 v2 beadchip uniquely mapped SNPs (all and discordant).**
(RAR)

Data S3 **BovineHD beadchip uniquely mapped SNPs (all and discordant).**
(RAR)

Data S4 **Affymetrix mouse diversity genotyping array uniquely mapped SNPs (all and discordant).**
(RAR)

Data S5 **Affymetrix Genome-Wide Human SNP Array 6.0 uniquely mapped SNPs (all and discordant).**
(RAR)

Data S6 **Illumina Human1M-Duo DNA Analysis beadchip uniquely mapped SNPs (all and discordant).**
(RAR)

Data S7 **Unix and R commands used to post-process the aligned sequences.**
(TXT)
